# Bis(1,3-benzothia­zole-2-thiol­ato)[(*Z*)-methyl 2-(2-amino-1,3-thia­zol-4-yl)-2-(methoxy­imino)acetate]nickel(II)

**DOI:** 10.1107/S1600536810015072

**Published:** 2010-04-30

**Authors:** Islam Ullah Khan, Onur Şahin, Shehzada Muhammad Sajid Jillani, Shahzad Sharif, Orhan Büyükgüngör

**Affiliations:** a Materials Chemistry Laboratory, Department of Chemistry, Government College University, Lahore 54000, Pakistan; b Department of Physics, Ondokuz Mayıs University, TR-55139 Samsun, Turkey

## Abstract

In the title compound, [Ni(C_7_H_4_NS_2_)_2_(C_7_H_9_N_3_O_3_S)], the Ni^II^ ion is in a slightly distorted N_4_S_2_ octa­hedral coordination environment. The two benzothia­zole-2-thiol­ate ligands chelate *via* their thia­zole N and thiol­ate S atoms while the methyl 2-(2-amino­thia­zol-4-yl)-2-(methoxy­imino)acetate also acts as a chelate ligand binding through the thia­zole and imino N atoms. Intra­molecular N—H⋯N, C—H⋯N and C—H⋯O inter­actions contribute to the mol­ecular conformation. In the crystal structure, inter­molecular N—H⋯O hydrogen bonds produce *R*_1_^2^(6) rings and generate chains along the *c* axis. An extensive one-dimensional supra­molecular network of N—H⋯O hydrogen bonds and C—H⋯π inter­actions is responsible for the crystal structure stabilization.

## Related literature

For the graph-set analysis of hydrogen-bond patterns, see: Bernstein *et al.* (1995 ▶). For related structures, see: Batı *et al.* (2006 ▶); Sieroń (2007 ▶); Liu & Xu (2004 ▶); Sharif *et al.* (2009 ▶); Song *et al.* (2005 ▶); Tashpulatov *et al.* (1957 ▶).
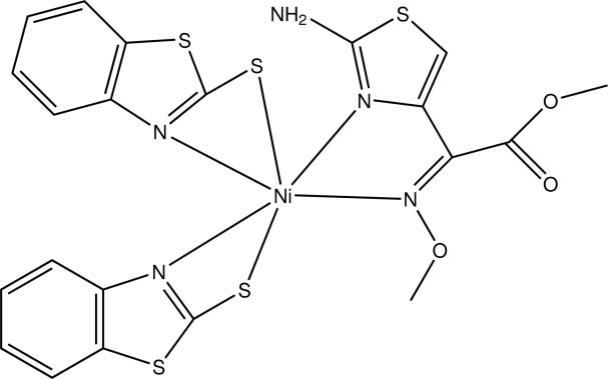


## Experimental

### 

#### Crystal data

[Ni(C_7_H_4_NS_2_)_2_(C_7_H_9_N_3_O_3_S)]
*M* *_r_* = 606.41
Monoclinic, 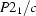

*a* = 17.8387 (11) Å
*b* = 7.8701 (5) Å
*c* = 17.9861 (10) Å
β = 98.639 (2)°
*V* = 2496.5 (3) Å^3^
*Z* = 4
Mo *K*α radiation
μ = 1.23 mm^−1^
*T* = 296 K
0.42 × 0.37 × 0.34 mm

#### Data collection

Bruker APEXII CCD area-detector diffractometer
26981 measured reflections
6164 independent reflections
3512 reflections with *I* > 2σ(*I*)
*R*_int_ = 0.056

#### Refinement

*R*[*F* ^2^ > 2σ(*F* ^2^)] = 0.043
*wR*(*F* ^2^) = 0.131
*S* = 1.03
6164 reflections
324 parameters
2 restraints
H atoms treated by a mixture of independent and constrained refinement
Δρ_max_ = 0.39 e Å^−3^
Δρ_min_ = −0.32 e Å^−3^

### 

Data collection: *APEX2* (Bruker, 2007 ▶); cell refinement: *SAINT* (Bruker, 2007 ▶); data reduction: *SAINT*; program(s) used to solve structure: *SHELXS97* (Sheldrick, 2008 ▶); program(s) used to refine structure: *SHELXL97* (Sheldrick, 2008 ▶); molecular graphics: *ORTEP-3 for Windows* (Farrugia, 1997 ▶); software used to prepare material for publication: *WinGX* (Farrugia, 1999 ▶).

## Supplementary Material

Crystal structure: contains datablocks global, I. DOI: 10.1107/S1600536810015072/sj2774sup1.cif

Structure factors: contains datablocks I. DOI: 10.1107/S1600536810015072/sj2774Isup2.hkl

Additional supplementary materials:  crystallographic information; 3D view; checkCIF report

## Figures and Tables

**Table 1:** Selected bond lengths (Å)

N1—Ni1	2.103 (3)
N2—Ni1	2.108 (2)
N3—Ni1	2.042 (3)
N4—Ni1	2.153 (3)
S2—Ni1	2.5410 (11)
S4—Ni1	2.5123 (10)

**Table 2:** Hydrogen-bond geometry (Å, °) *Cg*1 and *Cg*2 are the centroids of the C1–C6 and C8–C13 benzene rings, respectively.

*D*—H⋯*A*	*D*—H	H⋯*A*	*D*⋯*A*	*D*—H⋯*A*
N5—H5*A*⋯N2	0.85 (2)	2.25 (3)	3.036 (5)	154 (4)
N5—H5*B*⋯O2^i^	0.86 (2)	2.24 (3)	3.025 (5)	150 (4)
N5—H5*B*⋯O1^i^	0.86 (2)	2.38 (3)	3.036 (4)	133 (3)
C16—H16⋯O3	0.93	2.40	2.898 (4)	114
C21—H21*B*⋯N1	0.96	2.41	3.282 (5)	151
C4—H4⋯*Cg*2^ii^	0.93	2.93	3.588 (6)	129
C9—H9⋯*Cg*1^i^	0.93	2.99	3.636 (4)	128
C21—H21*A*⋯*Cg*2^iii^	0.96	2.76	3.556 (4)	141

Symmetry codes: (i) 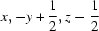; (ii) 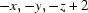; (iii) 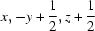.

**Fig. 1.**
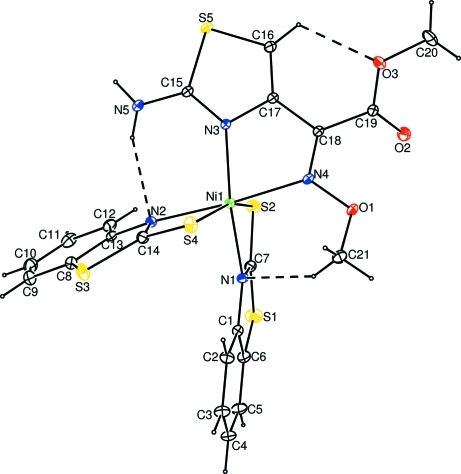
A view of the molecule of (I), showing the atom-numbering scheme. Displacement ellipsoids are drawn at the 30% probability level and H atoms are shown as small spheres of arbitrary radii. Hydrogen bonds are indicated by dashed lines.

**Fig. 2.**
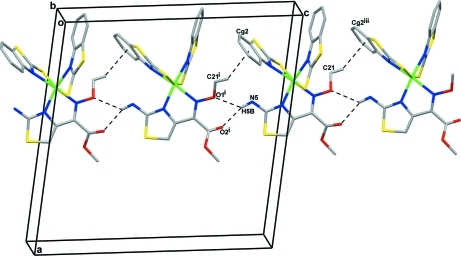
Part of the crystal structure of (I), showing the formation of R12(6) and R22(11) rings. N—H···O hydrogen bonds and C—H···π interactions are indicated by dashed lines. H atoms not involved in these interactions have been omitted for clarity. (Symmetry codes as in Table 2.)

**Fig. 3.**
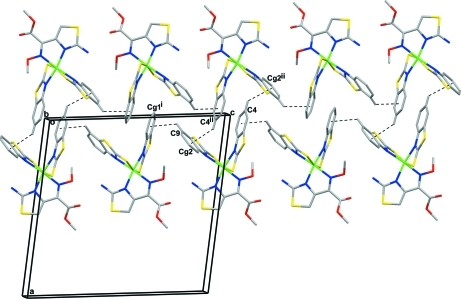
Part of the crystal structure of (I), showing the formation of a chain along [001] generated by the C—H···π interactions (dashed lines; see Table 2). For the sake of clarity, H atoms not involved in the motif shown have been omitted. (Symmetry codes as in Table 2.)

## supplementary crystallographic information

### Comment

This work was performed to explore the ligand properties of (2Z)-Methyl 2-(2-amino-1,3-thiazol-4-yl)-2-(methoxyimino)ethanoate (Sharif *et al.*,  2009), one of the precursors of *S*-1,3-Benzothiazol-2-yl(2Z)-2-(2-amino-1,3-thiazol-4-yl)-2- (methoxyimino)-ethanethioate (MAEM).   A study of the ligand behaviour of a second MAEM component 2-mercapto-benzothiazole has also been reported (Tashpulatov *et al.*,  1957).  We report here the structure of the title compound, (I), in which hydrogen bonds and C—H···π interactions lead to a one-dimensional supramolecular network.

The molecular structure of (I) and atom-labelling scheme are shown in Fig. 1. The Ni^II^ ion is coordinated by two S atoms (S2 and S4) and four N atoms (N1, N2, N3 and N4). The geometry around the Ni^II^ion is that of a slightly distorted octahedron. The title compound (I) has got two chelate ring types. In the first of these, atoms N3 and N4 are bonded to Ni1, thus generating five-membered chelate ring (C17/N3/Ni1/N4/C18). The other type, atoms N1, N2, S2 and S4 are bonded to Ni1 to form four-membered chelate rings (N1/Ni1/S2/C7) and (N2/Ni1/S4/C14). The two Ni—S distances are 2.5123 (10) and 2.5410 (11) Å. The Ni—N_thiazol_ distance of 2.042 (3)Å in (I) is almost equal to that in [Ni(NCS)(C_6_H_6_N_4_S_2_)(CH_4_O)]Cl (Liu and Xu, 2004). The Ni—N_benzothiazol_ distances in (I) are longer than the equivalent Pd—N bond distance (Song *et al.*, 2005), Co—N bond distances (Batı *et al.*, 2006) and Cu—N bond distance (Sieroń, 2007). The N1—C7 and N2—C14 bond lengths are indicative of significant double-bond character. These values are comparable with those found for similar compounds (Batı *et al.*, 2006; Sieroń, 2007). The N4═C18 and C19═O2 bond lengths are 1.281 (4) and 1.194 (4) Å, respectively, and agree with the corresponding distances in *S*-1,3-Benzothiazol-2-yl-(2Z)-2-(2-amino-1,3-thiazol-4-yl)-2-\ (methoxyimino)- ethanethioate [1.281 (3) and 1.190 (3) Å, respectively; Sharif *et al.*, 2009]. The C15—N3 bond is somewhat shorter than the C17—N3 bond, as a result of pronounced delocalization in the -N—C═N- fragment of the 2-aminothiazole ring. Each benzothiazole ligand is planar; the angles between the mean planes through the five-and six-membered rings of each ligand being 2.18 (14)° and 1.37 (20)°.

The molecules of (I) are linked by intermolecular hydrogen bonding, and we employ graph-set notation (Bernstein *et al.*, 1995) to describe the patterns of hydrogen bonding. Molecules of (I) are linked into sheets by a combination of N—H···O hydrogen bonds (Table 2). Within the selected asymmetric unit, intramolecular N—H···N, C—H···N and C—H···O hydrogen bonds define S(6) motifs (Fig. 1). Amino atom N5 in the reference molecule at (x, y, z) acts as hydrogen-bond donor, via atom H5B, to atom O1 in the molecule at (x, 1/2-y, z-1/2), so forming a C(7) chain running parallel to the [00-1] direction. Similarly, amino atom N5 in the reference molecule at (x, y, z) acts as hydrogen-bond donor, via atom H5B, to atom O2 in the molecule at (x, 1/2-y, z-1/2), so forming a C(8) chain running parallel to the [00-1] direction. The combination of C(7) and C(8) chains generates a chain of edge-fused R_1_^2^(6) rings running parallel to the [001] direction (Fig. 2).

Compound (I) also contains three intermolecular C—H···π interactions. In the first, atom C21 in the molecule at (x, y, z) acts as hydrogen-bond donor to the C8—C13 benzene ring in the molecule at (x, 1/2-y, 1/2+z), so forming a C(8) chain running parallel to the [001] direction. The combination of N—H···O hydrogen bonds and C—H···π interactions produce R_2_^2^(11) rings (Fig. 2). In the second, atom C4 in the molecule at (x, y, z) acts as hydrogen-bond donor to the C8—C13 benzene ring in the molecule at (-x, -y, 2-z), so forming a centrosymmetric R_2_^2^(20) rings centred at (0, 0, n+1) (n = zero or integer) (Fig. 3). Finally, atom C9 in the molecule at (x, y, z) acts as hydrogen-bond donor to the C1—C6 benzene ring in the molecule at (x, 1/2-y, z-1/2), so forming a C(9) chain running parallel to the [00-1] direction. Details of these interactions are given in Table 2. The combination of C—H···π interactions generates a chain of edge-fused R_2_^2^(20) and R_6_^6^(30) rings running parallel to the [001] direction (Fig. 3).

### Experimental

MAEM (0.25 g, 0.71 mmol) was dissolved in 20 ml methanol and refluxed for 10 minutes. A solution of nickel acetate (0.25 g, 0.18 mmol) previously prepared in 5 ml methanol was added dropwise, the mixture was refluxed further for one hour. The resulting solution was filtered. The filtrate was kept for slow evaporation, after three days light yellow crystals suitable for crystallography were obtained.

### Refinement

All H-atoms bound to C were refined using a riding model with d(C—H) = 0.93Å (U_iso_=1.2U_eq_ of the parent atom) for aromatic carbon atoms and d(C—H) = 0.96Å (U_iso_=1.5U_eq_ of the parent atom) for methyl carbon atoms. Amino H atoms were located in difference maps and refined subject to the DFIX restraint N—H = 0.87 (2) Å.

### Crystal data

**Table:**

[Ni(C_7_H_4_NS_2_)_2_(C_7_H_9_N_3_O_3_S)]	*F*(000) = 1240
*M**_r_* = 606.41	*D*_x_ = 1.613 Mg m^−^^3^
Monoclinic, *P*2_1_/*c*	Mo *K*α radiation, λ = 0.71073 Å
Hall symbol: -P 2ybc	Cell parameters from 4277 reflections
*a* = 17.8387 (11) Å	θ = 2.3–23.3°
*b* = 7.8701 (5) Å	µ = 1.23 mm^−^^1^
*c* = 17.9861 (10) Å	*T* = 296 K
β = 98.639 (2)°	Needle, colourless
*V* = 2496.5 (3) Å^3^	0.42 × 0.37 × 0.34 mm
*Z* = 4	

### Data collection

**Table:**

Bruker APEXII CCD area-detector diffractometer	3512 reflections with *I* > 2σ(*I*)
Radiation source: fine-focus sealed tube	*R*_int_ = 0.056
graphite	θ_max_ = 28.3°, θ_min_ = 1.2°
φ and ω scans	*h* = −23→23
26981 measured reflections	*k* = −10→10
6164 independent reflections	*l* = −20→23

### Refinement

**Table:**

Refinement on *F*^2^	Primary atom site location: structure-invariant direct methods
Least-squares matrix: full	Secondary atom site location: difference Fourier map
*R*[*F*^2^ > 2σ(*F*^2^)] = 0.043	Hydrogen site location: inferred from neighbouring sites
*wR*(*F*^2^) = 0.131	H atoms treated by a mixture of independent and constrained refinement
*S* = 1.03	*w* = 1/[σ^2^(*F*_o_^2^) + (0.0609*P*)^2^] where *P* = (*F*_o_^2^ + 2*F*_c_^2^)/3
6164 reflections	(Δ/σ)_max_ = 0.001
324 parameters	Δρ_max_ = 0.39 e Å^−^^3^
2 restraints	Δρ_min_ = −0.31 e Å^−^^3^

### Special details

**Table:**

Geometry. All esds (except the esd in the dihedral angle between two l.s. planes) are estimated using the full covariance matrix. The cell esds are taken into account individually in the estimation of esds in distances, angles and torsion angles; correlations between esds in cell parameters are only used when they are defined by crystal symmetry. An approximate (isotropic) treatment of cell esds is used for estimating esds involving l.s. planes.
Refinement. Refinement of *F*^2^ against ALL reflections. The weighted *R*-factor wR and goodness of fit *S* are based on *F*^2^, conventional *R*-factors *R* are based on *F*, with *F* set to zero for negative *F*^2^. The threshold expression of *F*^2^ > σ(*F*^2^) is used only for calculating *R*-factors(gt) etc. and is not relevant to the choice of reflections for refinement. *R*-factors based on *F*^2^ are statistically about twice as large as those based on *F*, and *R*- factors based on ALL data will be even larger.

### Fractional atomic coordinates and isotropic or equivalent isotropic displacement parameters (Å^2^)

**Table:**

	*x*	*y*	*z*	*U*_iso_*/*U*_eq_	
C1	0.1273 (2)	0.1742 (5)	1.06227 (18)	0.0477 (9)	
C2	0.0859 (2)	0.3229 (6)	1.0511 (2)	0.0597 (11)	
H2	0.1078	0.4225	1.0367	0.072*	
C3	0.0109 (3)	0.3189 (8)	1.0620 (3)	0.0794 (15)	
H3	−0.0182	0.4171	1.0540	0.095*	
C4	−0.0216 (3)	0.1725 (9)	1.0845 (3)	0.0903 (18)	
H4	−0.0722	0.1741	1.0916	0.108*	
C5	0.0185 (3)	0.0264 (8)	1.0963 (3)	0.0841 (16)	
H5	−0.0040	−0.0717	1.1115	0.101*	
C6	0.0942 (2)	0.0261 (6)	1.0853 (2)	0.0607 (11)	
C7	0.2253 (2)	−0.0043 (5)	1.06449 (18)	0.0443 (9)	
C8	0.1283 (2)	0.1743 (5)	0.80170 (18)	0.0440 (9)	
C9	0.0844 (2)	0.0802 (6)	0.7465 (2)	0.0593 (11)	
H9	0.0446	0.1301	0.7146	0.071*	
C10	0.1009 (2)	−0.0861 (6)	0.7403 (2)	0.0646 (12)	
H10	0.0720	−0.1508	0.7032	0.078*	
C11	0.1593 (3)	−0.1627 (5)	0.7872 (2)	0.0602 (11)	
H11	0.1693	−0.2774	0.7810	0.072*	
C12	0.2033 (2)	−0.0723 (5)	0.8431 (2)	0.0474 (9)	
H12	0.2420	−0.1248	0.8753	0.057*	
C13	0.18813 (18)	0.0992 (4)	0.84986 (16)	0.0342 (7)	
C14	0.20073 (18)	0.3672 (4)	0.89389 (18)	0.0396 (8)	
C15	0.4252 (2)	0.2366 (5)	0.90876 (18)	0.0449 (9)	
C16	0.5309 (2)	0.2658 (5)	1.01051 (19)	0.0511 (10)	
H16	0.5764	0.2740	1.0431	0.061*	
C17	0.46199 (18)	0.2752 (4)	1.03122 (17)	0.0395 (8)	
C18	0.44057 (19)	0.3070 (5)	1.10545 (17)	0.0400 (8)	
C19	0.4991 (2)	0.3044 (5)	1.17575 (19)	0.0480 (10)	
C20	0.6241 (2)	0.3713 (7)	1.2288 (2)	0.0851 (16)	
H20A	0.6677	0.4288	1.2156	0.128*	
H20B	0.6367	0.2549	1.2407	0.128*	
H20C	0.6085	0.4257	1.2718	0.128*	
C21	0.2788 (2)	0.4476 (6)	1.1692 (2)	0.0766 (14)	
H21A	0.2668	0.4729	1.2183	0.115*	
H21B	0.2407	0.3734	1.1434	0.115*	
H21C	0.2801	0.5511	1.1412	0.115*	
N1	0.20268 (14)	0.1531 (4)	1.05239 (14)	0.0379 (7)	
N2	0.22816 (14)	0.2105 (3)	0.90121 (14)	0.0341 (6)	
N3	0.40088 (14)	0.2583 (4)	0.97356 (14)	0.0390 (7)	
N4	0.36948 (16)	0.3292 (4)	1.10589 (14)	0.0421 (7)	
N5	0.3796 (2)	0.2109 (6)	0.84349 (18)	0.0689 (11)	
H5A	0.3330 (13)	0.195 (6)	0.846 (3)	0.099 (18)*	
H5B	0.400 (2)	0.197 (5)	0.8034 (15)	0.075 (14)*	
O1	0.35067 (14)	0.3667 (4)	1.17628 (12)	0.0565 (7)	
O2	0.48854 (16)	0.2341 (4)	1.23206 (15)	0.0793 (10)	
O3	0.56225 (14)	0.3783 (4)	1.16549 (13)	0.0603 (7)	
S1	0.15776 (7)	−0.14012 (16)	1.09195 (7)	0.0752 (4)	
S2	0.31353 (5)	−0.06091 (13)	1.04963 (6)	0.0561 (3)	
S3	0.12298 (6)	0.38890 (12)	0.82287 (6)	0.0565 (3)	
S4	0.24041 (6)	0.52105 (12)	0.95328 (5)	0.0520 (3)	
S5	0.52295 (5)	0.23624 (15)	0.91477 (5)	0.0567 (3)	
Ni1	0.29632 (2)	0.24588 (5)	1.00641 (2)	0.03629 (14)	

### Atomic displacement parameters (Å^2^)

**Table:**

	*U*^11^	*U*^22^	*U*^33^	*U*^12^	*U*^13^	*U*^23^
C1	0.035 (2)	0.077 (3)	0.0312 (18)	0.0005 (19)	0.0061 (15)	0.0008 (18)
C2	0.038 (2)	0.086 (3)	0.055 (2)	0.015 (2)	0.0075 (18)	−0.003 (2)
C3	0.042 (3)	0.134 (5)	0.065 (3)	0.022 (3)	0.018 (2)	−0.010 (3)
C4	0.035 (3)	0.180 (6)	0.059 (3)	0.001 (3)	0.022 (2)	−0.014 (3)
C5	0.049 (3)	0.143 (5)	0.067 (3)	−0.024 (3)	0.028 (2)	0.000 (3)
C6	0.045 (2)	0.095 (3)	0.045 (2)	−0.013 (2)	0.0139 (18)	0.002 (2)
C7	0.041 (2)	0.057 (2)	0.0363 (18)	−0.0005 (17)	0.0095 (15)	0.0108 (16)
C8	0.043 (2)	0.052 (2)	0.0368 (19)	−0.0017 (17)	0.0051 (16)	0.0007 (16)
C9	0.051 (2)	0.079 (3)	0.045 (2)	−0.006 (2)	−0.0011 (18)	−0.004 (2)
C10	0.069 (3)	0.074 (3)	0.051 (2)	−0.021 (2)	0.011 (2)	−0.019 (2)
C11	0.080 (3)	0.045 (2)	0.061 (3)	−0.016 (2)	0.031 (2)	−0.009 (2)
C12	0.055 (2)	0.043 (2)	0.047 (2)	−0.0021 (18)	0.0171 (18)	0.0041 (17)
C13	0.0340 (18)	0.042 (2)	0.0288 (16)	−0.0045 (15)	0.0114 (13)	0.0041 (14)
C14	0.0390 (19)	0.042 (2)	0.0379 (18)	0.0000 (16)	0.0066 (14)	0.0059 (15)
C15	0.0353 (19)	0.070 (3)	0.0310 (17)	0.0007 (17)	0.0116 (14)	0.0010 (17)
C16	0.035 (2)	0.084 (3)	0.0359 (18)	0.0064 (19)	0.0094 (15)	0.0042 (18)
C17	0.0318 (18)	0.058 (2)	0.0298 (16)	0.0000 (15)	0.0078 (13)	0.0029 (15)
C18	0.0311 (18)	0.063 (2)	0.0272 (16)	0.0015 (16)	0.0076 (13)	0.0020 (15)
C19	0.035 (2)	0.081 (3)	0.0286 (19)	0.0021 (18)	0.0049 (15)	0.0021 (17)
C20	0.041 (2)	0.140 (5)	0.067 (3)	−0.006 (3)	−0.019 (2)	0.011 (3)
C21	0.047 (3)	0.131 (4)	0.054 (2)	0.021 (3)	0.014 (2)	−0.028 (3)
N1	0.0304 (15)	0.0493 (18)	0.0359 (15)	0.0019 (13)	0.0111 (11)	0.0052 (13)
N2	0.0316 (15)	0.0374 (16)	0.0335 (14)	−0.0010 (12)	0.0052 (11)	0.0032 (12)
N3	0.0300 (15)	0.062 (2)	0.0258 (13)	−0.0044 (13)	0.0061 (11)	−0.0009 (13)
N4	0.0345 (16)	0.067 (2)	0.0276 (14)	0.0020 (14)	0.0120 (12)	−0.0021 (13)
N5	0.043 (2)	0.136 (4)	0.0301 (17)	−0.014 (2)	0.0131 (15)	−0.0117 (19)
O1	0.0418 (15)	0.102 (2)	0.0273 (12)	0.0106 (14)	0.0117 (10)	−0.0078 (13)
O2	0.0513 (18)	0.146 (3)	0.0385 (16)	−0.0071 (17)	0.0009 (13)	0.0277 (17)
O3	0.0345 (15)	0.099 (2)	0.0440 (14)	−0.0096 (14)	−0.0039 (11)	0.0060 (14)
S1	0.0697 (8)	0.0732 (8)	0.0864 (8)	−0.0135 (6)	0.0235 (6)	0.0291 (7)
S2	0.0450 (6)	0.0578 (6)	0.0653 (6)	0.0135 (5)	0.0076 (5)	0.0124 (5)
S3	0.0585 (7)	0.0500 (6)	0.0549 (6)	0.0118 (5)	−0.0118 (5)	0.0046 (5)
S4	0.0647 (7)	0.0398 (5)	0.0495 (5)	−0.0027 (5)	0.0023 (5)	−0.0028 (4)
S5	0.0347 (5)	0.1025 (9)	0.0359 (5)	0.0064 (5)	0.0150 (4)	0.0007 (5)
Ni1	0.0291 (2)	0.0506 (3)	0.0298 (2)	0.0005 (2)	0.00674 (16)	0.00160 (19)

### Geometric parameters (Å, °)

**Table:**

C1—C2	1.382 (6)	C15—N3	1.315 (4)
C1—N1	1.392 (4)	C15—N5	1.340 (4)
C1—C6	1.398 (5)	C15—S5	1.730 (4)
C2—C3	1.380 (6)	C16—C17	1.339 (5)
C2—H2	0.9300	C16—S5	1.722 (4)
C3—C4	1.378 (8)	C16—H16	0.9300
C3—H3	0.9300	C17—N3	1.394 (4)
C4—C5	1.354 (7)	C17—C18	1.464 (5)
C4—H4	0.9300	C18—N4	1.281 (4)
C5—C6	1.394 (6)	C18—C19	1.514 (4)
C5—H5	0.9300	C19—O2	1.194 (4)
C6—S1	1.724 (5)	C19—O3	1.305 (4)
C7—N1	1.311 (4)	C20—O3	1.463 (4)
C7—S2	1.695 (4)	C20—H20A	0.9600
C7—S1	1.737 (4)	C20—H20B	0.9600
C8—C9	1.383 (5)	C20—H20C	0.9600
C8—C13	1.401 (4)	C21—O1	1.421 (4)
C8—S3	1.737 (4)	C21—H21A	0.9600
C9—C10	1.350 (6)	C21—H21B	0.9600
C9—H9	0.9300	C21—H21C	0.9600
C10—C11	1.377 (6)	N1—Ni1	2.103 (3)
C10—H10	0.9300	N2—Ni1	2.108 (2)
C11—C12	1.376 (5)	N3—Ni1	2.042 (3)
C11—H11	0.9300	N4—O1	1.389 (3)
C12—C13	1.385 (5)	N4—Ni1	2.153 (3)
C12—H12	0.9300	N5—H5A	0.848 (19)
C13—N2	1.390 (4)	N5—H5B	0.863 (19)
C14—N2	1.326 (4)	S2—Ni1	2.5410 (11)
C14—S4	1.697 (3)	S4—Ni1	2.5123 (10)
C14—S3	1.747 (3)		
			
C2—C1—N1	126.0 (4)	C17—C18—C19	121.1 (3)
C2—C1—C6	120.5 (4)	O2—C19—O3	125.2 (3)
N1—C1—C6	113.4 (4)	O2—C19—C18	122.5 (3)
C3—C2—C1	117.9 (5)	O3—C19—C18	112.2 (3)
C3—C2—H2	121.0	O3—C20—H20A	109.5
C1—C2—H2	121.0	O3—C20—H20B	109.5
C4—C3—C2	121.4 (5)	H20A—C20—H20B	109.5
C4—C3—H3	119.3	O3—C20—H20C	109.5
C2—C3—H3	119.3	H20A—C20—H20C	109.5
C5—C4—C3	121.3 (5)	H20B—C20—H20C	109.5
C5—C4—H4	119.3	O1—C21—H21A	109.5
C3—C4—H4	119.3	O1—C21—H21B	109.5
C4—C5—C6	118.7 (5)	H21A—C21—H21B	109.5
C4—C5—H5	120.7	O1—C21—H21C	109.5
C6—C5—H5	120.7	H21A—C21—H21C	109.5
C5—C6—C1	120.2 (4)	H21B—C21—H21C	109.5
C5—C6—S1	129.3 (4)	C7—N1—C1	111.8 (3)
C1—C6—S1	110.5 (3)	C7—N1—Ni1	98.7 (2)
N1—C7—S2	119.5 (3)	C1—N1—Ni1	148.0 (2)
N1—C7—S1	114.8 (3)	C14—N2—C13	112.0 (3)
S2—C7—S1	125.7 (2)	C14—N2—Ni1	96.95 (19)
C9—C8—C13	120.9 (4)	C13—N2—Ni1	148.1 (2)
C9—C8—S3	129.4 (3)	C15—N3—C17	110.3 (3)
C13—C8—S3	109.6 (2)	C15—N3—Ni1	133.5 (2)
C10—C9—C8	118.2 (4)	C17—N3—Ni1	115.8 (2)
C10—C9—H9	120.9	C18—N4—O1	114.2 (3)
C8—C9—H9	120.9	C18—N4—Ni1	115.5 (2)
C9—C10—C11	121.8 (4)	O1—N4—Ni1	128.5 (2)
C9—C10—H10	119.1	C15—N5—H5A	116 (3)
C11—C10—H10	119.1	C15—N5—H5B	118 (3)
C12—C11—C10	121.1 (4)	H5A—N5—H5B	125 (4)
C12—C11—H11	119.4	N4—O1—C21	110.6 (2)
C10—C11—H11	119.4	C19—O3—C20	116.0 (3)
C11—C12—C13	118.1 (3)	C6—S1—C7	89.47 (19)
C11—C12—H12	121.0	C7—S2—Ni1	74.11 (13)
C13—C12—H12	121.0	C8—S3—C14	90.12 (16)
C12—C13—N2	125.8 (3)	C14—S4—Ni1	74.26 (11)
C12—C13—C8	119.8 (3)	C16—S5—C15	89.61 (17)
N2—C13—C8	114.4 (3)	N3—Ni1—N1	160.83 (11)
N2—C14—S4	119.2 (2)	N3—Ni1—N2	100.11 (10)
N2—C14—S3	113.8 (2)	N1—Ni1—N2	85.53 (10)
S4—C14—S3	126.9 (2)	N3—Ni1—N4	76.10 (10)
N3—C15—N5	123.9 (3)	N1—Ni1—N4	101.37 (10)
N3—C15—S5	114.1 (2)	N2—Ni1—N4	169.15 (11)
N5—C15—S5	121.9 (3)	N3—Ni1—S4	100.16 (8)
C17—C16—S5	110.2 (3)	N1—Ni1—S4	98.95 (8)
C17—C16—H16	124.9	N2—Ni1—S4	68.27 (7)
S5—C16—H16	124.9	N4—Ni1—S4	102.12 (8)
C16—C17—N3	115.8 (3)	N3—Ni1—S2	93.47 (8)
C16—C17—C18	129.7 (3)	N1—Ni1—S2	67.44 (8)
N3—C17—C18	114.4 (3)	N2—Ni1—S2	100.15 (7)
N4—C18—C17	115.0 (3)	N4—Ni1—S2	90.28 (9)
N4—C18—C19	123.8 (3)	S4—Ni1—S2	163.39 (4)
			
N1—C1—C2—C3	178.5 (3)	C1—C6—S1—C7	0.4 (3)
C6—C1—C2—C3	−1.3 (5)	N1—C7—S1—C6	0.9 (3)
C1—C2—C3—C4	1.0 (6)	S2—C7—S1—C6	−176.7 (3)
C2—C3—C4—C5	−0.4 (7)	N1—C7—S2—Ni1	−5.0 (2)
C3—C4—C5—C6	0.0 (7)	S1—C7—S2—Ni1	172.4 (2)
C4—C5—C6—C1	−0.2 (6)	C9—C8—S3—C14	−178.4 (4)
C4—C5—C6—S1	−177.0 (4)	C13—C8—S3—C14	0.4 (3)
C2—C1—C6—C5	0.9 (6)	N2—C14—S3—C8	−0.3 (3)
N1—C1—C6—C5	−178.9 (3)	S4—C14—S3—C8	−178.2 (3)
C2—C1—C6—S1	178.2 (3)	N2—C14—S4—Ni1	−10.3 (2)
N1—C1—C6—S1	−1.6 (4)	S3—C14—S4—Ni1	167.6 (3)
C13—C8—C9—C10	−0.1 (6)	C17—C16—S5—C15	−0.5 (3)
S3—C8—C9—C10	178.6 (3)	N3—C15—S5—C16	0.4 (3)
C8—C9—C10—C11	0.3 (6)	N5—C15—S5—C16	−177.5 (4)
C9—C10—C11—C12	0.4 (6)	C15—N3—Ni1—N1	100.0 (4)
C10—C11—C12—C13	−1.4 (6)	C17—N3—Ni1—N1	−71.8 (4)
C11—C12—C13—N2	−177.8 (3)	C15—N3—Ni1—N2	−5.8 (4)
C11—C12—C13—C8	1.6 (5)	C17—N3—Ni1—N2	−177.5 (2)
C9—C8—C13—C12	−0.9 (5)	C15—N3—Ni1—N4	−175.4 (4)
S3—C8—C13—C12	−179.8 (3)	C17—N3—Ni1—N4	12.9 (2)
C9—C8—C13—N2	178.5 (3)	C15—N3—Ni1—S4	−75.3 (3)
S3—C8—C13—N2	−0.4 (4)	C17—N3—Ni1—S4	113.0 (2)
S5—C16—C17—N3	0.5 (4)	C15—N3—Ni1—S2	95.2 (3)
S5—C16—C17—C18	−176.9 (3)	C17—N3—Ni1—S2	−76.6 (2)
C16—C17—C18—N4	171.8 (4)	C7—N1—Ni1—N3	−8.9 (4)
N3—C17—C18—N4	−5.7 (5)	C1—N1—Ni1—N3	−170.9 (4)
C16—C17—C18—C19	−11.0 (6)	C7—N1—Ni1—N2	99.2 (2)
N3—C17—C18—C19	171.5 (3)	C1—N1—Ni1—N2	−62.8 (4)
N4—C18—C19—O2	41.7 (6)	C7—N1—Ni1—N4	−89.2 (2)
C17—C18—C19—O2	−135.2 (4)	C1—N1—Ni1—N4	108.7 (4)
N4—C18—C19—O3	−141.9 (4)	C7—N1—Ni1—S4	166.38 (19)
C17—C18—C19—O3	41.1 (5)	C1—N1—Ni1—S4	4.3 (4)
S2—C7—N1—C1	175.8 (2)	C7—N1—Ni1—S2	−3.72 (18)
S1—C7—N1—C1	−1.9 (4)	C1—N1—Ni1—S2	−165.8 (4)
S2—C7—N1—Ni1	5.9 (3)	C14—N2—Ni1—N3	−104.5 (2)
S1—C7—N1—Ni1	−171.79 (17)	C13—N2—Ni1—N3	99.9 (4)
C2—C1—N1—C7	−177.5 (3)	C14—N2—Ni1—N1	94.0 (2)
C6—C1—N1—C7	2.3 (4)	C13—N2—Ni1—N1	−61.6 (4)
C2—C1—N1—Ni1	−16.7 (6)	C14—N2—Ni1—N4	−36.0 (6)
C6—C1—N1—Ni1	163.1 (3)	C13—N2—Ni1—N4	168.4 (5)
S4—C14—N2—C13	178.3 (2)	C14—N2—Ni1—S4	−7.52 (18)
S3—C14—N2—C13	0.1 (4)	C13—N2—Ni1—S4	−163.1 (4)
S4—C14—N2—Ni1	11.9 (3)	C14—N2—Ni1—S2	160.08 (19)
S3—C14—N2—Ni1	−166.23 (18)	C13—N2—Ni1—S2	4.5 (4)
C12—C13—N2—C14	179.5 (3)	C18—N4—Ni1—N3	−16.6 (3)
C8—C13—N2—C14	0.1 (4)	O1—N4—Ni1—N3	179.9 (3)
C12—C13—N2—Ni1	−26.7 (6)	C18—N4—Ni1—N1	144.0 (3)
C8—C13—N2—Ni1	153.9 (3)	O1—N4—Ni1—N1	−19.6 (3)
N5—C15—N3—C17	177.6 (4)	C18—N4—Ni1—N2	−87.2 (6)
S5—C15—N3—C17	−0.2 (4)	O1—N4—Ni1—N2	109.2 (6)
N5—C15—N3—Ni1	5.6 (6)	C18—N4—Ni1—S4	−114.2 (3)
S5—C15—N3—Ni1	−172.25 (18)	O1—N4—Ni1—S4	82.2 (3)
C16—C17—N3—C15	−0.2 (5)	C18—N4—Ni1—S2	76.9 (3)
C18—C17—N3—C15	177.6 (3)	O1—N4—Ni1—S2	−86.6 (3)
C16—C17—N3—Ni1	173.4 (3)	C14—S4—Ni1—N3	102.97 (14)
C18—C17—N3—Ni1	−8.8 (4)	C14—S4—Ni1—N1	−75.46 (14)
C17—C18—N4—O1	−177.3 (3)	C14—S4—Ni1—N2	6.05 (15)
C19—C18—N4—O1	5.6 (5)	C14—S4—Ni1—N4	−179.22 (15)
C17—C18—N4—Ni1	16.7 (4)	C14—S4—Ni1—S2	−41.67 (19)
C19—C18—N4—Ni1	−160.4 (3)	C7—S2—Ni1—N3	−178.75 (14)
C18—N4—O1—C21	159.1 (3)	C7—S2—Ni1—N1	2.95 (14)
Ni1—N4—O1—C21	−37.2 (4)	C7—S2—Ni1—N2	−77.83 (14)
O2—C19—O3—C20	0.5 (6)	C7—S2—Ni1—N4	105.16 (14)
C18—C19—O3—C20	−175.8 (4)	C7—S2—Ni1—S4	−33.55 (19)
C5—C6—S1—C7	177.4 (4)		

### Hydrogen-bond geometry (Å, °)

**Table:**

Cg1 and Cg2 are the centroids of the C1–C6 and C8–C13 benzene rings, respectively.

**Table:**

*D*—H···*A*	*D*—H	H···*A*	*D*···*A*	*D*—H···*A*
N5—H5A···N2	0.85 (2)	2.25 (3)	3.036 (5)	154 (4)
N5—H5B···O2^i^	0.86 (2)	2.24 (3)	3.025 (5)	150 (4)
N5—H5B···O1^i^	0.86 (2)	2.38 (3)	3.036 (4)	133 (3)
C16—H16···O3	0.93	2.40	2.898 (4)	114
C21—H21B···N1	0.96	2.41	3.282 (5)	151
C4—H4···Cg2^ii^	0.93	2.93	3.588 (6)	129
C9—H9···Cg1^i^	0.93	2.99	3.636 (4)	128
C21—H21A···Cg2^iii^	0.96	2.76	3.556 (4)	141

Symmetry codes: (i) *x*, −*y*+1/2, *z*−1/2; (ii) −*x*, −*y*, −*z*+2; (iii) *x*, −*y*+1/2, *z*+1/2.
